# A Systematic Review and Meta-Analysis on a Disease in TCM: Astragalus Injection for Gathering Qi Depression

**DOI:** 10.1155/2020/2803478

**Published:** 2020-02-12

**Authors:** Yanxiang Ha, Po Huang, Yumeng Yan, Xiaolong Xu, Bo Li, Yuhong Guo, Qingquan Liu

**Affiliations:** ^1^Beijing Hospital of Traditional Chinese Medicine, Capital Medical University, Beijing Institute of Traditional Chinese Medicine, Beijing 100010, China; ^2^Beijing University of Chinese Medicine, Beijing 100029, China

## Abstract

Zong Qi depression is a disease recorded in the literature of Chinese traditional medicine for a long time. In recent years, the theory of Zong Qi depression has been more and more applied to the diagnosis and treatment of a variety of diseases. Astragalus is the most important drug used to treat the depression of Zong Qi. Meanwhile, Astragalus injection is also widely used in a variety of diseases in accordance with the manifestations of Zong Qi subsidence. However, there is a lack of systematic review or meta-analysis of the clinical effect of Astragalus injection in the treatment of Zong Qi subsidence. Therefore, we searched for diseases characterized by symptoms of Zong Qi subsidence (including heart failure, respiratory failure, acute respiratory distress syndrome, and acute lung injury) and evaluated the effect of Astragalus injection in these diseases with mortality and distance of a 6-minute walking test. The results showed that the mortality of patients with subsidence of Zong Qi decreased in 1 month (OR, 0.26 [0.12, 0.61], 95% CI, *P*=0.002) and 1 year (OR, 0.38 [0.20, 0.69], 95% CI, *P*=0.002) after using Astragalus injection. The distance of 6-minute walking test after 7 (MD, 91.60 [6.89, 176.31], 95% CI, *P*=0.03), 14 (MD, 22.62 [13.80, 31.43], 95% CI, *P* < 0.00001), and 28 days (MD, 108.31 [30.02, 186.59], 95% CI, *P*=0.007) of using Astragalus injection also increased. Therefore, we believe that Astragalus injection has a certain therapeutic effect on the depression of Zong Qi.

## 1. Introduction

**Figure 1:**
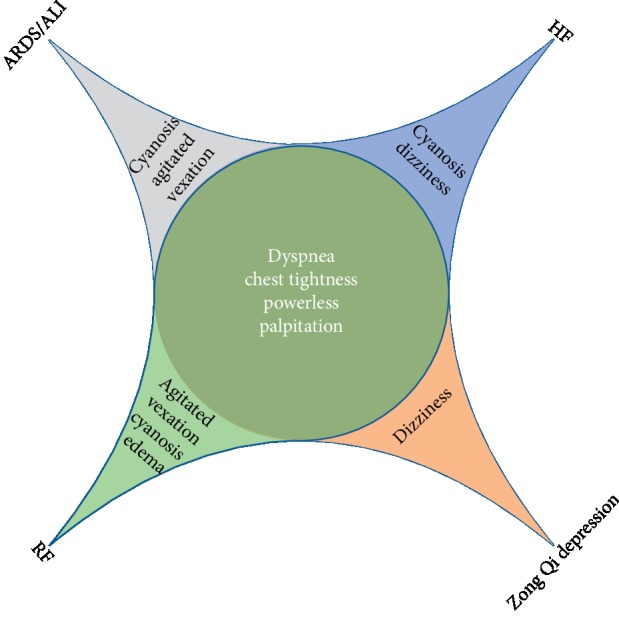
In the system of syndrome differentiation and treatment of traditional Chinese medicine, HF, RF, ARDS, and ALI belong to Zong Qi depression.

Qi is a subtle substance in human body in traditional Chinese medicine (TCM) theory. As one of the leading substances, the function of Qi covers almost all aspects of human function. Qi deficiency causes pale face, shortness of breath, weakness of limbs, dizziness, diarrhea, and other symptoms. Qi deficiency is associated with a variety of diseases, especially cardiovascular and cerebrovascular diseases [1, 2]. According to the guidance of TCM, the use of *Atractylodes macrocephala*, Astragalus, and even medical qigong has played a therapeutic effect on many diseases caused by Qi deficiency [3–5]. Gathering Qi, one of the Qi in human body, known as Zong Qi or “Da Qi” frequently, is first mentioned in “Huang Di Nei Jing:” “Five cereals which enters the stomach were divided into three portions: dross, essence, and gathering Qi. Gathering Qi is accumulated in the chest, out of the throat,” and its function is “active heart to push blood as well as lung to breath.” There are many supplements in the generations, Yu Jiayan, one of famous doctors lived in the Qing dynasty, believe that gathering Qi can dominate a patient whether to survive [6]. And, Zhang Xichun proposed that gathering Qi has a close relationship with general health, and it is a series of complicated symptoms when gathering Qi dysfunction, such as shortness of breath, asthma, chest tightness, fatigue, and palpitation, is the most widely described description. While the gathering Qi depression, or Zong Qi depression, usually has the same clinical symptoms but more severe, dyspnea is a common symptom in acute and critical illness. Zhang Xichun also proposed a treatment while perfecting the theory: Shengxian decoction, the most famous gecoction of traditional Chinese medicine for the treatment of Zong Qi depression. The composition of Shengxian decoction is as follows: *Astragalus membranaceus* (huangqi), *Anemarrhena* (zhimu), Radix Bupleuri (chaihu), Rhizoma cohosh (shengma), and *Platycodon grandiflorum* (jiegeng). This decoction is widely used to treat the symptoms of Zong Qi deficiency characterized by fatigue and shortness of breath [7, 8]. *Astragalus membranaceus* plays a dominate role and described by Zhang Xichun as “best at filling the Da Qi in the chest.” Pharmacological studies have shown that Astragalus and its extracts showed immunostimulant, antioxidative, and cardioprotective effects [9, 10]. All these are attributed to the function of Astragalus in increasing Zong Qi. However, in the study of Astragalus, there is a little emphasis on Zong Qi depression rather than specific diseases in modern medicine. This condition hinders our assessment of the role of Astragalus. Thus, we regarded sinking of gathering Qi as an independent disease and corresponded to western medicine, founded it is similar to many disease and closest to heart failure (HF), respiratory failure (RF), acute respiratory distress syndrome (ARDS), and acute lung injury (ALI). The above 4 diseases cannot completely represent the suffocation, but they have typical clinical symptoms (Figure 1). Astragalus injection is made of a sterilized extraction solution from Astragalus and widely used in clinical practice [11]. Similar to Astragalus decoction, the main components of Astragalus injection are astragalosides, polysaccharides, flavones, and amino acids [12]. In a meta-analysis study, both Astragalus injection and Astragalus granule have achieved certain curative effects [13]. However, because the drug in the form of injection can play a directly role without oral absorption, there is a better curative effect of uningested chemicals in the blood [14]. So far, no research has been done to unify the above diseases into Zong Qi depression; hence, we searched for clinical trials of Astragalus injection for the treatment of them and conducted systematic and meta-analysis to exploring the therapeutic effect of Astragalus injection on diseases with Zong Qi depression.

## 2. Methods

A systematic review and meta-analysis of clinical studies assessed the efficacy of Astragalus injection combined with routine therapy for Zong Qi depression which includes heart failure, respiratory failure, acute respiratory distress syndrome, and acute lung injury. We divided the mortality into short-term (less than 1 month) [15] and long-term (1 month to 1 year) [16], and the distance of 6-minute walking test after continuous use of Astragalus injection for 7 days, 14 days, and 28 days for evaluation of therapeutic effect in order to evaluate the therapeutic effect of Astragalus injection.

### 2.1. Search Strategy

We conducted a literature search on Pubmed, China National Knowledge Infrastructure (CNKI), WanFang Data, and Vip citation databases from inception to March 19, 2019. Search strategy and details are follows: (Cardiac Failure or Heart failure or Shock Lung or Acute Respiratory Distress Syndrome or ARDS or ALI or Acute Lung Injury or Respiratory Insufficiency or Respiratory Failure) and (Astragalus injection or Huangqi injection). Research was carried out on filtering out nonclinical trials during search.

### 2.2. Study Selection

This study of meta-analysis was to assessing the therapeutic effect of Astragalus injection for Zong Qi depression. The primary outcomes were mortality which is divided into 1-month mortality and 1-year mortality. The secondary outcome indicator is distance of 6-minute walk test (6MWT). The following inclusion criteria were required for a study to be eligible for the meta-analysis: (1) a clinical study, at least one disease of heart failure, respiratory failure, acute respiratory distress syndrome, or acute lung injury, as long as the diagnosis of the above four diseases, regardless of which version of the clinical practice guide; (2) a study conducted in a population aged 18 years or older; (3) a study including the use of Astragalus injection without other Chinese medicine or Chinese medicine extracts;and (4) a study including report mortality or 6-minute walk test. We excluded reviews or studies on animals.

### 2.3. Data Extraction

Retrieved studies were selected and appraised by two trained reviewers (YH and YY). Disagreement were discussed and decided with a third reviewer (PH). After that, two independent reviewers (YH and YY) extracted data from the included studies. For each eligible study, we extracted data on the name of researcher and year of publication, diseases reported in the study, sample size including the number of subjects in the experimental group and control group, participant characteristics (age and sex), course of disease, routine therapy, application methods of Astragalus injection, Outcome indicators, and side effects.

### 2.4. Statistical Methods

We conducted this meta-analysis to calculate the therapeutic effect of Astragalus injection on the mortality of patients with Zong Qi depression using a fixed-effects model. The random effect model was used to calculate the therapeutic effect of Astragalus injection on the 6-minute walk test. The effect of selection of binary variables is RR value, while that of continuous variables is mean difference. Point estimates and 95% confidence intervals (CI) of each effect were calculated. Heterogeneity test statistical method uses *Q* test, gets probability by *χ*^2^ analysis, and quantitatively describes the degree of heterogeneity by *I*^2^ value to evaluate the size of heterogeneity. Sensitivity analysis is done by omitting one report at a time from the analysis and assessing its impact on overall results. Furthermore, the potential publication bias was analyzed by funnel plots. The data extracted from the literature were analyzed by Revman 5.3, and a 2-sided *P* value less than 0.05 was considered statistically significant.

The quality of clinical studies was assessed according to the Cochrane Collaboration's Tool for Assessing Risk of Bias (ROB table). This scale awards nine scores to each study as follows: random sequence generation, distribution methods, participant blindness, blindness of outcome evaluation, incomplete outcome data, selective reporting, and other biases, including the description of these seven items, including “high risk,” “low risk,” and “unclear risk” in the three judgements, and the risk assessment results are represented by a bias risk map. Based on the results of this systematic review, use the GRADE system to recommend a grading recommendation method to evaluate the quality of evidence. Divide the quality of the indicators included in this study into high: further research will not change the credibility of the results; medium: further research may alter the reliability and outcome of the treatment; low: further studies are likely to alter the reliability and outcome of the treatment; and very low: any evaluation of efficacy and results are uncertain. The assessment results are presented in the summary of findings table (SoF table).

## 3. Results

### 3.1. Study Selection

**Figure 2:**
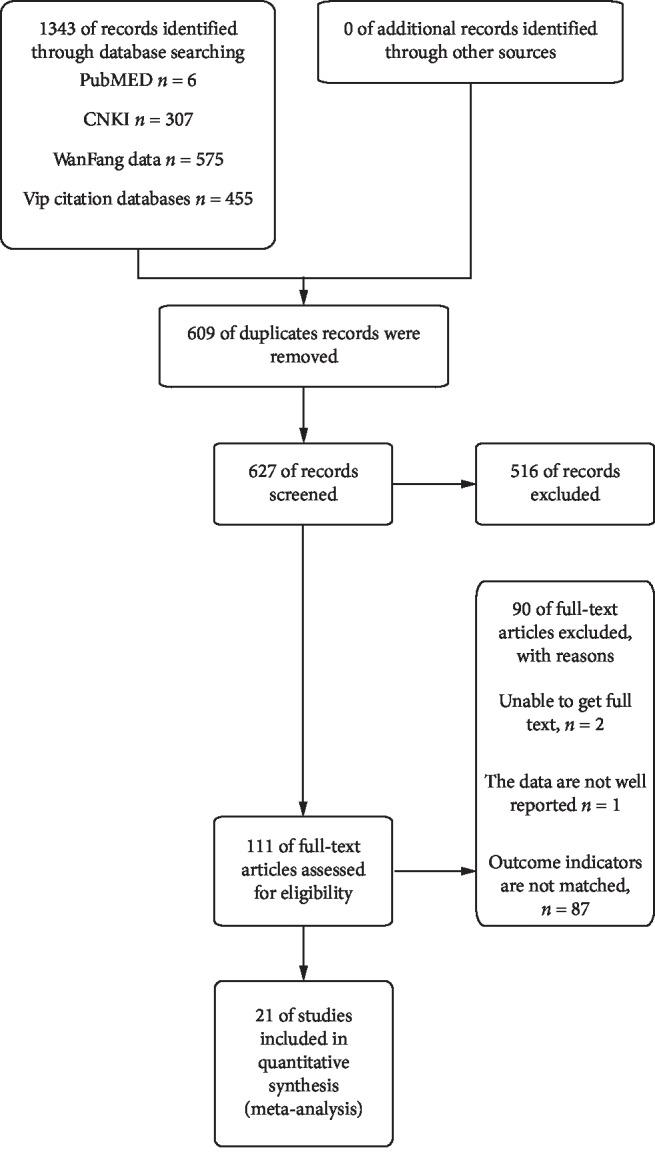
Study flow diagram: after searching, a total of 1343 articles were found, 21 of which met the criteria, and were included.

From the searches for systematic reviews, 1343 records were identified including PubMED (*n* = 6), CNKI (*n* = 307), WanFang Data (*n* = 575), and Vip citation databases (*n* = 455). After excluding 609 duplicate studies, 627 were excluded based on title/abstract contents. The full texts of 111 records were read, and 21 studies involving 1868 participants were ultimately included (Figure 2).

### 3.2. Study Characteristics

A total of 21 eligible studies involving 1868 participants were included in this study, including one reported on ARDS [17], one on respiratory failure [18], 18 on heart failure [19–36], and one on both of respiratory failure and heart failure [37]. Of the 18 reported heart failure studies, four reported pulmonary heart disease as a comorbidity [22, 26, 28, 29], and the others are chronic or congestive heart failure ([Supplementary-material supplementary-material-1] provided in Supplementary Materials). All 21 studies were conducted China.

Studies on HF included used a variety of diagnostic criteria, mainly including ACC/AHA 2005 Guideline Update for the Diagnosis and Management of Chronic Heart Failure in the Adult [38], Framingham criteria [39], and Guidelines for the diagnosis and management of chronic heart failure which was made by Chinese Society of Cardiology of Chinese Medical Association [40]. According to these clinical practice guidelines, cardiotonic, diuretic, vasodilator, and oxygen inhalation are used to control the primary disease and eliminate the inducement of heart failure. The other three diseases are also diagnosed and treated according to the corresponding clinical practice guidelines [41].

### 3.3. Outcomes

#### 3.3.1. Primary and Secondary Outcomes

**Figure 3:**
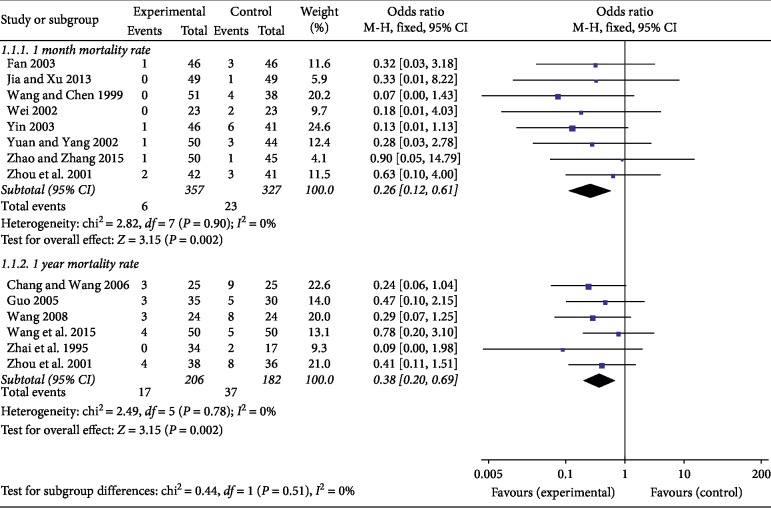
Based on fixed-effect model, the use of Astragalus injection can reduce the one-month mortality and one-year mortality of patients with Zong Qi depression.

In reporting mortality rate as an outcome indicator 13 studies, 8 studies [18, 21, 26, 28, 29, 33, 36, 37] including 684 patients reported a one-month mortality rate, and 6 studies [17, 19, 20, 25, 30, 36] including 388 patients reported a one-year mortality rate. The use of Astragalus injection can reduce the one-month mortality (OR, 0.26 [0.12, 0.61], 95% CI, *P*=0.002) and one-year mortality (OR, 0.38 [0.20, 0.69], 95% CI, *P*=0.002) of patients with Zong Qi depression (Figure 3). Both of two indicators have a low heterogeneity (*P*=0.90, *I*^2^ = 0% and *P*=0.78, *I*^2^ = 0%).

**Figure 4:**
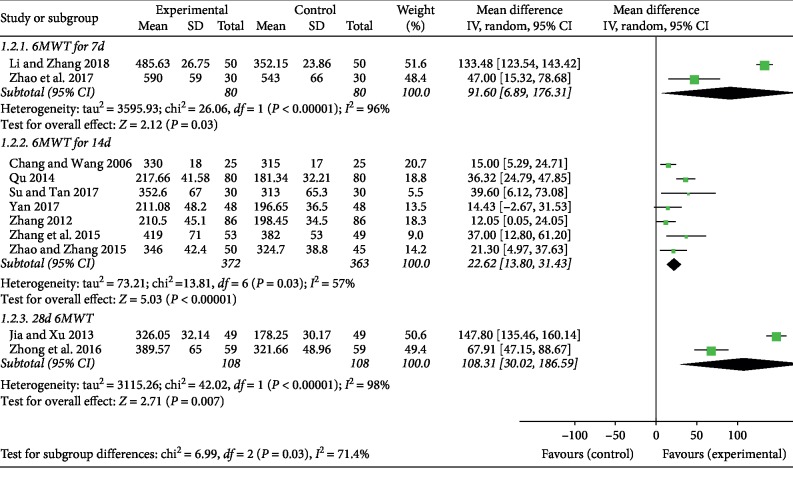
After 7 days, 14 days, and 28 days of Astragalus injection, the distance of 6-minute walking test increased.

Two studies [22, 34] including 160 patients reported 6-minute walking distance after 7 days with Astragalus injection, seven studies [19, 23, 24, 27, 31–33] including 735 patients reported 14 days, and two studies [21, 35] including 216 patients for 28 days. Pooled estimates showed that the distance of 6-minute walking test increased (MD, 91.60 [6.89, 176.31], 95% CI, *P*=0.03) after 7 days with Astragalus injection, as well as 14 days (MD, 22.62 [13.80, 31.43], 95% CI, *P* < 0.00001) and 28 days (MD, 108.31 [30.02, 186.59], 95% CI, *P*=0.007), Figure 4. Among these indicators, the heterogeneity between studies is greater, which is *P* < 0.00001, *I*^2^ = 96%, *P*=0.03, *I*^2^ = 57% and *P* < 0.007, *I*^2^ = 98%. The reasons for this high heterogeneity will be discussed in Section 4.

#### 3.3.2. Side Effect

One patient developed dizziness, two patients developed feeling of fullness in the head, one patient had elevated serum urea nitrogen, three patients had first dose hypotension, one patient had hypotension, one patient had sinus bradycardia, and one patient had second degree atrioventricular block in all included studies. Among them, some patients can alleviate by themselves, and some have been alleviated by reducing dosage or symptomatic treatment.

#### 3.3.3. Sensitivity Analyses

In sensitivity analysis, we found that excluding any study would not significantly change the results.

#### 3.3.4. Bias Risk Assessment

**Figure 5:**
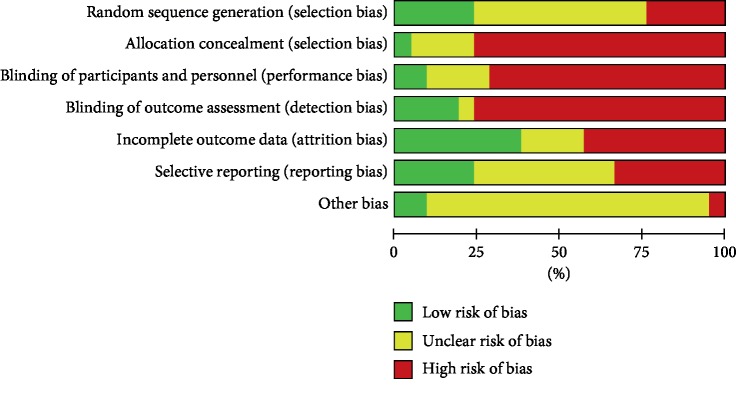
Risk of bias graph reviews judgements about each risk of bias item presented as percentages across all included 21 studies.

**Figure 6:**
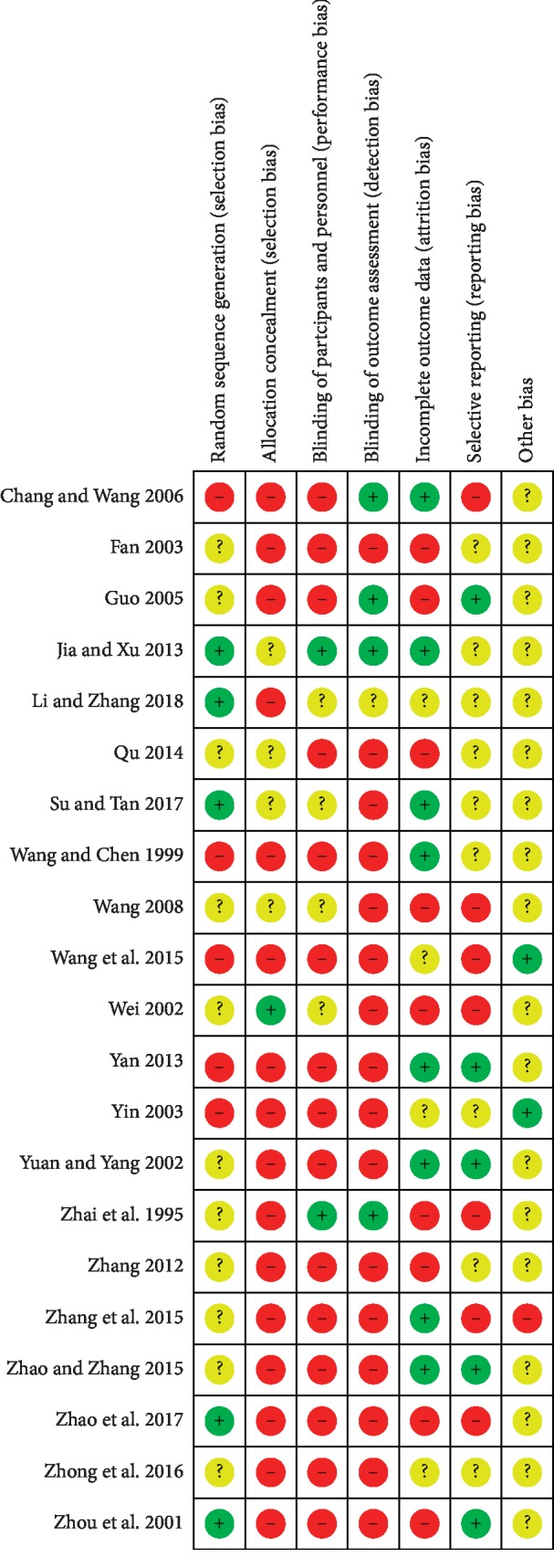
Risk of bias summary reviews authors' judgements about each risk of bias item for each included studies.

The assessment of ROB is summarized. The quality of most studies is relatively low. Most studies have mentioned the use of random allocation, but only a small number describe the way of random allocation, and almost no research reports allocation concealment. Concealment of subjects is feasible when injected with Astragalus injection, but no studies have reported it. All studies were unable to blind the interventions to participants (Figures 5 and 6).

**Figure 7:**
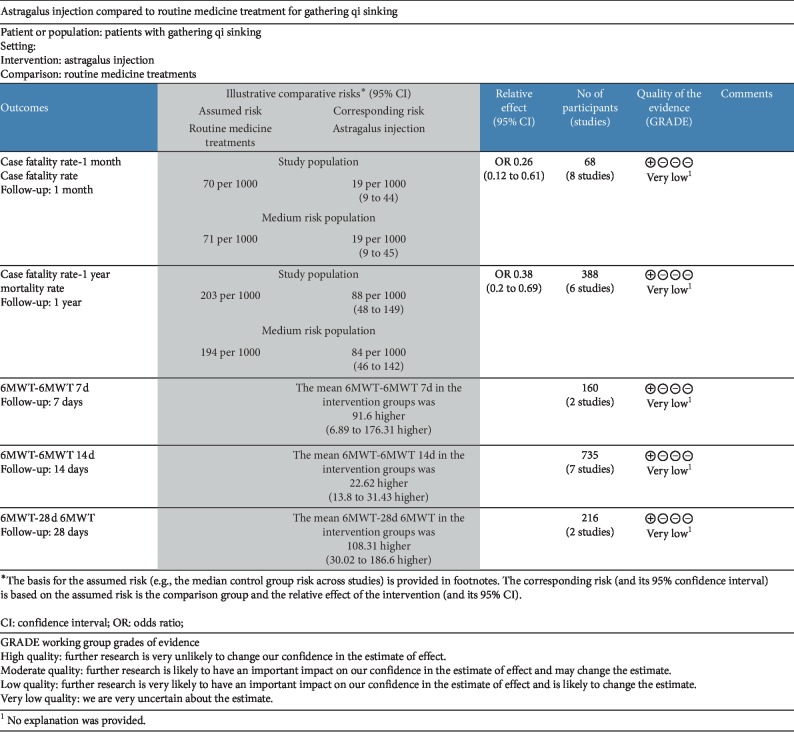
GRADE summary of findings table.

There were five main outcomes in this study. The mortality was followed up for one month and one year, and the walking distance of 6-minute walk test after 7 days, 14 days, and 28 days with Astragalus injection. The quality of evidence for each outcome is very low (Figure 7). The main reasons are unreported double-blind methods and large inconsistencies.

#### 3.3.5. Publication Bias

**Figure 8:**
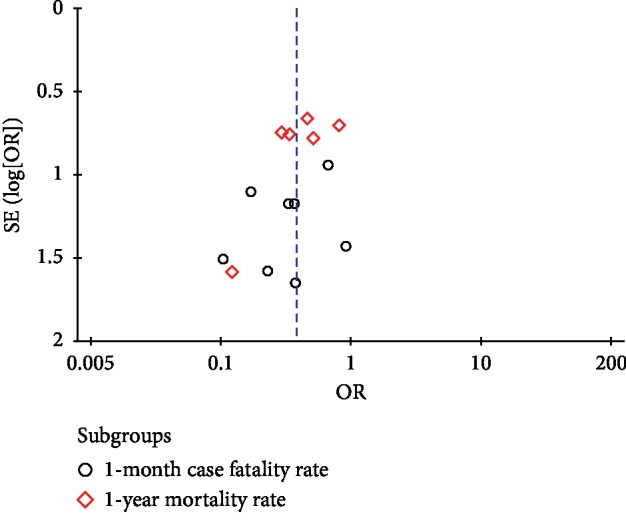
Astragalus injection vs. routine medicine treatments: outcome: mortality rate.

The funnel plots for publication bias show no obvious asymmetry, and it indicated that the pooled results were not influenced by the publication bias (Figure 8).

## 4. Discussion

In this meta-analysis, we searched clinical studies of Astragalus injection in the treatments of four diseases that accord with clinical feature of Zong Qi depression: heart failure, respiratory failure, acute respiratory distress syndrome, and acute lung injury and found that Astragalus injection combined with routine medicine treatments on gathering Qi depression can reduce the short-term mortality as well as long-term mortality. Furthermore, 7 days, 14 days, and 28 days continuous use of Astragalus injection can increase the 6-minute walking test of Zong Qi depression patients. These findings are supported by precious systematic review on heart failure [42]; however, the therapeutic of Astragalus injection on the other diseases are rarely studied. This shows that application scope of Astragalus injection still needs to be explored; therefore, we cautiously suggest that it can be considered combining Astragalus injection with routine treatment to reduce mortality and improve clinical efficacy when facing gathering Qi depression.

In traditional Chinese medicine theory, the lungs govern respiration which means they inhale “pure Qi” from air and exhale “dirty Qi,” the combination of air from the lungs and food Qi from the spleen forms gathering Qi which derives Yang Qi of the heart and the lung. Thus function of gathering Qi is closely related to the whole body condition and more closely to circulation and respiratory function, as Zhang Xichun said “gathering Qi actually controls general health while remains in cheat.” Like Qi deficiency, when there is insufficient of Zong Qi, we often show symptoms such as fatigue and shortness of breath, which leads to decreased activity tolerance [2, 43]. With the development of modern medicine, we found that the function of Zong Qi is related to the respiratory system, circulatory system, nervous system, and even reproductive system. And, the number of diseases that treated by the theory of Zong Qi is increasing, including but not limited to chronic congestive heart failure, coronary heart disease, senile cardiac arrhythmias, sinus bradycardia, vertebral basilar artery insufficiency, the complete atrioventricular block, frequent room premature beat, viral myocarditis, quickness, sick sinus syndrome, rheumatic heart disease, atrial fibrillation, cor pulmonale, bronchial asthma and other respiratory system diseases, circulatory system disease, and dizziness, neurosis, tension headaches, gastroptosis, abdominal distention, diarrhea, urinary incontinence, retention of urine, premature ejaculation, functional uterine bleeding, and other system diseases [44]. While gathering depression is a more serious stage, according to Zhang Xichun “people feel that exhalation and inhalation cannot be continuous, that means Da Qi is about to become weak,” we choose asthma as the main symptom of gathering Qi dysfunction and selected four diseases with most typical symptoms for systematic review and meta-analysis: heart failure, respiratory failure, acute respiratory distress syndrome, and acute lung injury. After the search, we only found and included 3 of them. In the literature included in this study, the symptoms of the patients included lethargy, cyanosis of lips, chest pain, cough and expectoration, dyspnea, palpitation, and edema. These clinical manifestations are consistent with the disease Zong Qi depression described by Zhang Xichun. It is precisely in his theory that Astragalus is the first choice for the treatment of Zong Qi depression. In previous studies, traditional Chinese medicine has achieved good results in the treatment of heart and lung diseases. When thinking that patients need to nourish Yang Qi, doctors often use drugs such as ginseng and aconite [45]. In recent years, *Astragalus membranaceus* has increasingly appeared in drugs used to treat heart and lung diseases, such as Qishen Yiqi dripping pills and Qili Qiangxin capsules [46, 47]. Therefore, we think it is necessary to evaluate the efficacy of Astragalus injection in the treatment of these diseases.

Obviously, this does not completely cover all the diseases contained in Zong Qi depression, and at the same time, some of the symptoms are not completely subordinate to it [48]. On the one hand, it is attributed to the fact that function of Zong Qi is too broad to be summarized clearly; on the other hand, it is also due to the lack of objective evaluation criteria for the concept of Qi deficiency. Zong Qi depression has been widely studied and discussed as a symptom, but this is far from the original intention of ancient Chinese medicine doctors.

In the selection of outcome indicators, our first consideration is the mortality rate. Meanwhile, 6-minute walking test is a gold indicator for assessing cardiopulmonary functions by test activity tolerance. Qi is considered to be the most essential substance in human beings, directly reflecting life activities and activity tolerance, and Astragalus is one of the first choices for Qi in TCM theory, especially good at relieving fatigue and improving activity tolerance [49]. In this study, Astragalus injection can reduce the mortality and increase the distance of 6-minutes walking test of patients with Zong Qi depression, which is in line with the theory of traditional Chinese medicine. In other clinical trials on respiratory and circulatory systems, Astragalus injection has also yielded good results [50–52].

There is no such dosage form of injection in traditional Chinese medicine theory. In the practice of Chinese doctors for thousands of years, drugs act on the human body in the form of water decoction. With the support of healthy atmosphere, the spleen and stomach work together to help the drug effectively become absorbed and play a therapeutic role. However, the decoction of traditional Chinese medicine has some weaknesses on solubility and stability [53]. Therefore, it is very important to develop new dosage forms for the application of Chinese herbal medicine [54]. At the same time, due to the standardization of the manufacture of traditional Chinese medicine, the difficulty of homogenization of quality and the complexity of components, the adverse reactions occurred frequently in the process of application. Therefore, it is necessary to strengthen the systematic research work of traditional Chinese medicine injection and the supervision and control of clinical rational drug use, and conduct high-quality clinical trials to clarify the use of traditional Chinese medicine injection [55].

For a long time, we have been using traditional Chinese medicine theory, in other words, treatment based on syndrome differentiation, to guide the use of traditional Chinese herbs to treat diseases in Western medicine. We seem to have been used to saying that treatment of chronic heart failure with Astragalus injection is under the theoretical guidance of Yi Qi Sheng Yang theory (a commonly used theory in Chinese medicine was proposed by Zhang Xichun). This will definitely affect our understanding and evaluation of the efficacy of traditional Chinese medicine to a certain extent. Therefore, we innovatively produced a systematic review which contains diseases in TCM theory. To our knowledge, this is the first meta-analysis to comprehensively summarize results regarding heart failure, respiratory failure, and acute respiratory distress syndrome as an independent disease; at the same time, it is also the first systematic review of treating diseases in TCM theory. We recommend take more similar studies to verify Chinese medicine theory and efficacy to change the current situation of diagnosis with western medicine and treatment with traditional Chinese medicine.

This study included 21 studies with 4 diseases, so a high heterogeneity is neither surprising nor avoidable. On the one hand, the dose of traditional Chinese medicine used varies greatly; on the other hand, the included patients suffer from different basic diseases. Such problems also exist in the other systematic reviews of TCM fields. This indicates that we need relevant studies on the usage and dosage of Chinese medicine to explore the best curative effect to guide clinical treatment and establish the corresponding regulations. We believe that it is necessary to establish and improve the system of disease names with Chinese medicine characteristics. Using TCM theory to guide the treatment of diseases in TCM theory may get better curative effect. There may be a long way to go, but we should start now. In addition, the quality of included studies was low, suggesting the need for high-quality clinical studies.

## Supplementary Materials

Supplementary Materials Table 1: basic information on the studies included.
