# Supplementary material for: A Systematic Review and Meta-Analysis on a Disease in TCM: Astragalus Injection for Gathering Qi Depression
Source: Evid Based Complement Alternat Med. 2020 Feb 12;2020:2803478. doi: 10.1155/2020/2803478 (PMC7038034; doi:10.1155/2020/2803478)
Supplement: Supplementary Materials — Table 1: basic information on the studies included. [file 2803478.f1.pdf]

Researcher name/year	Reported disease	Sample size (Experimental group/Control group)	Sex (male/ female)	age	Course of the disease	conventional therapy	Usage and dosage of Astragalus injection	Outcome indicators
**Li 2018**	Pulmonary heart disease with congestive heart failure	90 (45/45)	Experimental group 28/17 Control group 26/19	Experimental group 61.29±3.37 Control group 62.38±3.44	Experimental group 9.87±3.55 Control group 10.45±3.67 (month)	All patients were gaven guidance instructed to rest on time. Metoprolol was given twice a day, 6.25 mg each time for 15 days.	Astragalus injection was injected intravenously into 50 g/L glucose solution once a day, 40 ml each time for 15 days.	2
**Zhong 2016**	Chronic congestive heart failure	118(59/59)	Experimental group 35/27 Control group 32/27	Experimental group 66.27±3.41 Control group 66.43±3.52	Experimental group 3.14±1.13 Control group 3.26±1.19 (year)	Symptomatic supportive treatment such as bed rest, continuous low flow oxygen inhalation, cardiac tonicity, diuresis, vasodilation, and digitalis drugs should be used when necessary.	Astragalus injection 60 ml + saline 250 ml intravenous drip once a day for 28 days	2
**Wang 2016**	Acute respiratory distress syndrome	100(50/50)		Experimental group 43.4±4.10 Control group 43.4±4.60		Mechanical ventilation, anti-infection, liquid therapy	Astragalus injection 20 ml + saline to 100 ml intravenous drip once a day for 5 days	1
**Jia 2013**	Chronic heart failure	98(49/49)	Experimental group 23/26	Experimental group	Experimental group	Angiotensin-converting enzyme inhibitors (lisinopril tablets),	Astragalus injection, adding 5% glucose solution or 0.9% sodium chloride injection	1,2

			Control group 21/28	66.46±9.15 Control group 67.55±8.58	2.25±0.84 Control group 2.19±1.02 (year)	diuretics (hydrochlorothiazide tablets), digitalis preparations and other medicines were fixed and used simultaneously, and the dosage was adjusted according to the patient's condition.	250ml in slow intravenous drip, once a day, for 4 weeks	
**Zhang 2012**	Chronic congestive heart failure	172(86/86)	Experimental group 51/35 Control group 36/48	Experimental group 65.50±11.52 Control group 64.50±11.17	Experimental group 1-23 Control group 1-20 (year)	Comprehensive treatment of digitalis, diuretics and angiotensin converting enzyme inhibitors	Astragalus injection was added into 20ml glucose injection 250ml intravenous drip once a day for 14 days	2
**Chang 2006**	Chronic heart failure	50(25/25)	28/22	58.5	3.8 (year)	Conventional anti-CHF treatment, including diuretics, cardiac sputum (digoxigenin), angiotensin converting enzyme inhibitors, nitrates and the like. After 2 weeks of stable condition, add carvedilol	Intravenous infusion of 40ml of Astragalus injection, once a day, changed to daily jaundice 40g after 14 days, powdered into the end, the disease was stable for 2 weeks, plus carvedilol, the initial dose of 3.125mg, hemodynamic stability The heart rate is >60 beats/min, and the dose is incremented every 2 weeks, and each time is increased to 2 times of the upper dose until the target dose of 25 mg, 2 times/d or the maximum tolerated dose is maintained.	1,2
**Guo 2005**	Congestive heart failure	65(35/30)	Experimental group 21/14 Control	Experimental group 62.5 ±7. 2	Experimental group 2-15	Bed rest, a diet rich in vitamins and proteins, and avoidance of emotional agitation are all treated	Astragalus injection 60ml added with 5% glucose injection 250ml intravenously for 30 drops/min once a day for 14 days.	1

			group 18/21	Control group 57.25±5.1	Control group 1-18 (year)	according to CHF routine, including digitalis inhibitors, diuretics, ACEI, etc.		
**Zhai 1995**	Congestive heart failure	51(34/17)	Experimental group 23/11 Control group 12/5	Experimental group 38-80 Control group 41-79	Experimental group 6-36 Control group 6-36 (month)	Heart function class II, III, IV patients take Digoxin tablets 0.25mg, class IV patients plus furosemide 20mg once a day	20 ml of astragalus injection, 10% glucose injection 250ml intravenously once a day, 10 days	1
**Fan 2003**	Severe pulmonary heart disease with respiratory failure	92(46/46)	Experimental group 34/12 Control group 36/10	Experimental group 63. 6 ±6.8 Control group 64.5±7.2		Anti-infective, Asthma treatment expectorant, oxygen, cardiac diuretic, vasodilator and other conventional treatments	Astragalus injection 40ml plus 5% glucose intravenously, 1 time / d, 10-15d	1
**Yan 2013**	Chronic congestive heart failure	96(48/48)	Experimental group 25/23 Control group 25/23	Experimental group 65.35±10.50 Control group 65.35±10.56	Experimental group 1-20 Control group 1-20 (year)	Digitalis, diuretics, angiotensin converting enzyme inhibitors, etc.	Astragalus injection 20 ml force into 5% glucose injection 250 ml intravenously once a day for14 days	2
**Wei 2002**	heart failure from Pulmonary heart disease	46(23/23)	Experimental group 13/10 Control group 11/12	Experimental group 25/23 Control group 25/23	Experimental group 25/23 Control group 25/23	Comprehensive treatments such as anti-infection, oxygen therapy, improved ventilation, diuresis and symptomatic support. Patients with arrhythmia are given Antiarrhythmic treatment, and	40 ml of Astragalus injection was added to 10% glucose 250 ml intravenously, once a day for 7 to 10 days	1

						pay attention to correct water and electrolyte disorders, according to the condition plus the appropriate amount of digitalis drugs for treatment		
**Yuan 2002**	heart failure from Pulmonary heart disease	94(50/44)	50/44	50-70		Anti-infection, oxygen inhalation, cardiac tonicity, diuresis, improvement of ventilation function, etc.	Astragalus injection 12ml added 10% glucose solution 300ml intravenous drip once a day for14 days	1
**Su 2017**	chronic heart failure	60(30/30)	Experimental group 12/18 Control group 6/24	Experimental group 70.0±6.91 Control group 70.9±10.9		To control risk factors, diuretics, angiotensin-converting enzyme inhibitors (ACEI) or as appropriate Angiotensin receptor antagonists (ARB), beta-blockers, aldosterone antagonists and digitalis	Astragalus injection 20 mL dissolved in 5% glucose injection<br>Static point in 250 mL for 2 weeks	2
**Zhao 2017**	chronic heart failure	60(30/30)	24/36	56-85	3-18(year)	Heart strengthening, diuresis, vasodilation, oxygen inhalation, etc.	Astragalus injection 30 ml, once a day for 12 days.	2
**Yin 2003**	chronic heart failure from Pulmonary heart disease	87(46/41)	Experimental group 35/11 Control group 31/10	Experimental group 60.3 Control group 62.1		Routine oxygen inhalation, anti-infection, antiasthmatic and asthma relief, phlegm and cough relief and symptomatic treatment	Astragalus injection 30ml added with 5% glucose injection 250ml intravenous drip once a day, once a day, 1O for 15 days	1
**Wang 2008**	chronic heart failure	48(24/24)	Experimental group 13/11	Experimental group 48-75		Routine use of digitalis, diuretics and vasodilators	Astragalus injection (produced by Chengdu Di'ao Group) 40 ml intravenous drip once a	1

			Control group 14/10	Control group 46-73			day for 14 days	
**Zhang 2015**	heart failure	106（53/53）	Experimental group 30/23 Control group 31/22	Experimental group 67.2 ±5.8 Control group 65. 9 ± 5.3		Statin lipid-lowering, aspirin and/or clopidogrel antiplatelet, beta-blocker inhibiting sympathetic activity, ACEI or ARB drugs delaying ventricular remodeling, spironolactone inhibiting aldosterone receptor, furosemide diuretic reducing cardiac preload	Astragalus injection 20 mL + 5% glucose injection 250 mL static point, once a day for 14 days	2
**Zhao 2015**	chronic heart failure	93（51/46）	53/44	71.5 ± 8.7		Bed rest, oxygen inhalation, loop diuretics, angiotensin converting enzyme inhibitors or angiotensin II receptor antagonists, beta-blockers, positive inotropic drugs, vasodilators and other conventional anti-heart failure treatment, arrhythmia patients take amiodarone tablets	Astragalus injection 40 mL added 0.5% glucose injection 250 mL intravenous drip once a day for 14 days "	1,2
**Zhou 2001**	Congestive heart failure	83(41/41)	Experimental group 24/18 Control group 25/16	Experimental group 68 ±5 Control group 67 ±6	Experimental group 1-20 Control group 1-18 (year)	Conventional treatment of CHF includes digitalis preparation, diuretics and angiotensin converting enzyme inhibitors.	Astragalus injection 40 ml and 5% glucose injection 500 ml were intravenously dripped once a day for 14 days	1
**Qu 2014**	Congestive	160(80/80)	Experimental	Experimental	Experimental	Digoxin 0.125-0.25 mg once a	Astragalus injection 20 ml plus 5% glucose	2

	heart failure		group 42/38 Control group 43/37	group 60.55±11.4 Control group 60.45±11.4	group 1-20 Control group 1-20 (year)	day 121 times; Dihydrochlorothiazide tablet 25 mg and Spironolactone tablet 20 mg twice a day; Captopril 12.5-25 mg, 3 times a day. Patients with cardiac function grade IV received intravenous drip of sodium nitroprusside, furosemide, dopamine and dobutamine according to their condition. Corresponding symptomatic treatment was adopted for patients with complications.	injection 100 ml intravenous drip once a day for 14 days	
**Wang 1999**	Pulmonary heart disease with heart failure and respiratory failure	89(51/38)	Experimental group 31/21 Control group 23/15	group 58.3±11 Control group 60.2±13		Oxygen therapy, antibiotics, expectorant and asthma relief, correction of water and electrolyte, acid-base balance, diuresis and respiratory stimulants	Astragalus injection 40-60 ml added 250 ml saline intravenous drip	1
